# Circadian rhythm dysregulation and ferroptosis in hypertension: mechanisms and therapeutic implications

**DOI:** 10.3389/fcvm.2026.1889670

**Published:** 2026-10-16

**Authors:** Hui Zeng, Jiawen Lin, Jun Chen

**Affiliations:** 1 Department of Cardiology, Shanghai Yangpu Hospital of Traditional Chinese Medicine, Shanghai, China; 2 Faculty of Chinese Medicine, Macau University of Science and Technology, Macau, China

**Keywords:** blood vessel, Circadian rhythm, ferroptosis, hypertension, Kidney

## Abstract

Hypertension exhibits a pronounced circadian rhythm; however, the mechanisms by which circadian disruption translates temporal imbalance into sustained vascular and renal injury remain unclear. Increasing evidence suggests that circadian misalignment may promote endothelial dysfunction, vascular remodeling, and renal injury through ferroptosis driven by oxidative stress dysregulation, thereby increasing the risk of hypertension. Based on current evidence, this review proposes that circadian disruption induces a temporal mismatch between oxidative stress generation and antioxidant defense, thereby lowering the threshold for ferroptosis in cardiovascular and renal tissues. This process may amplify hemodynamic stress, impair endothelial integrity, and disturb renal sodium handling, ultimately contributing to the development and progression of hypertension. Although direct causal evidence remains limited, this review integrates circadian biology with ferroptosis and provides a mechanistic link between time-dependent physiological regulation and structural organ damage, highlighting a potentially promising strategy for future hypertension intervention.

## Graphical Abstract

**Figure:**
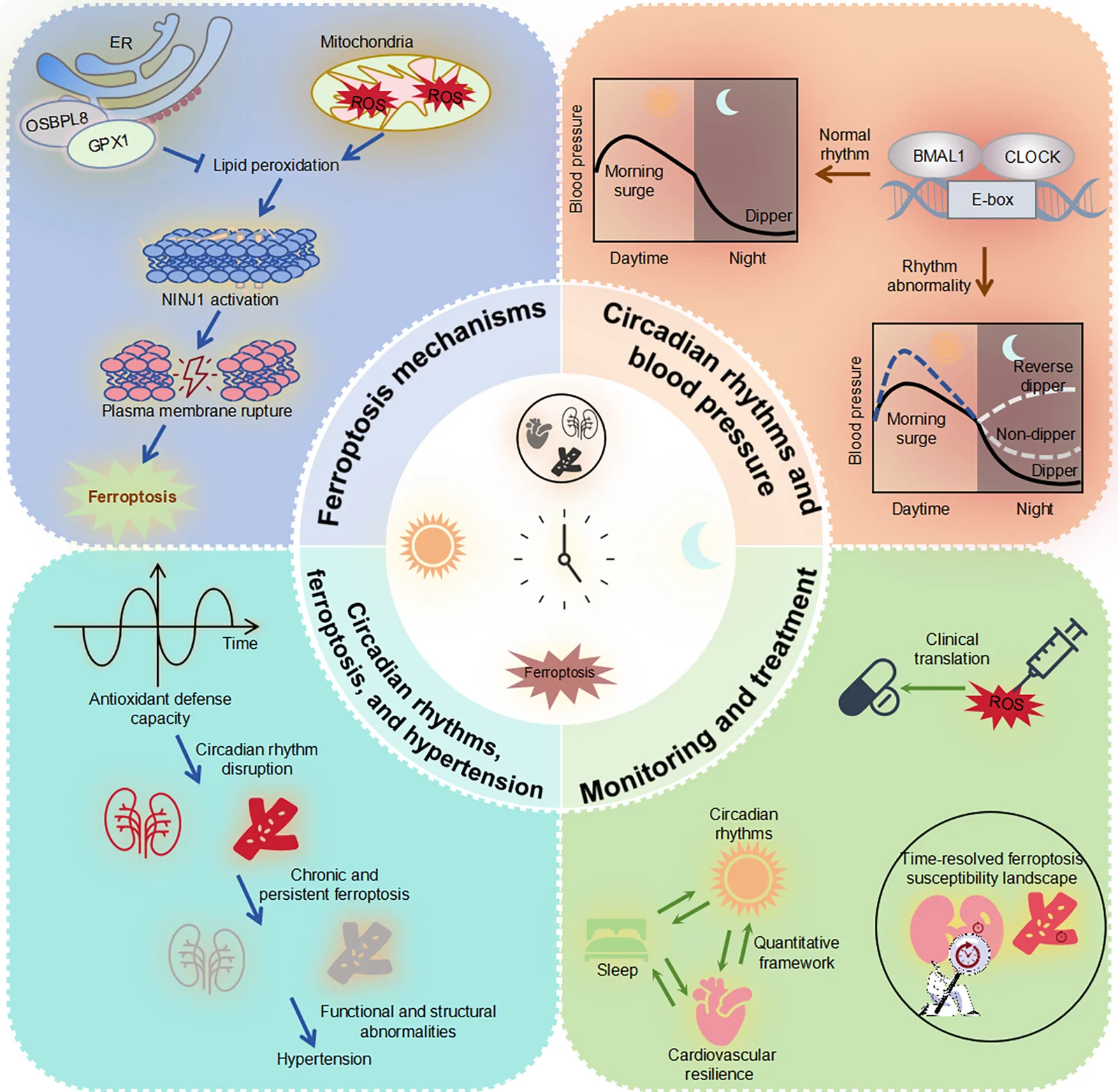

## 1 Introduction

The development of hypertension has traditionally been attributed to mechanisms such as neurohumoral activation, renal dysfunction, and vascular remodeling. However, these classical paradigms do not fully account for a fundamental physiological feature of blood pressure (BP) regulation-its pronounced circadian rhythmicity (1). Under physiological conditions, BP follows a dynamic pattern characterized by a nocturnal decline and a surge in the early morning, which is tightly regulated by the time-dependent coordination of sympathetic nervous system activity, vascular tone, and endocrine signaling (2). The nocturnal reduction in BP is accompanied by decreased mechanical stress on the vascular wall and attenuation of oxidative stress, both of which are essential for maintaining vascular elasticity and overall cardiovascular homeostasis (3).

Circadian rhythms are generated through the precise regulation of clock genes. Transcriptomic analyses have shown that more than 40% of protein-coding genes exhibit rhythmic expression in at least one tissue (4), including a substantial number of genes involved in the regulation of redox homeostasis (5, 6). This time-dependent oscillation in antioxidant capacity enables organisms to dynamically adapt to fluctuations in oxidative stress across the day-night cycle. However, when circadian rhythms are disrupted-such as by sleep deprivation or circadian misalignment—this finely tuned temporal coordination is impaired, leading to sustained oxidative stress, reduced capacity for lipid peroxide detoxification, and consequently increased susceptibility to cellular injury (6).

Ferroptosis is a form of regulated cell death driven by iron-dependent lipid peroxidation (7) and has recently been recognized as a critical contributor to vascular dysfunction and renal injury (8, 9). Vascular endothelial cells and smooth muscle cells, characterized by high oxidative metabolic activity and membranes enriched in polyunsaturated lipids, are particularly susceptible to ferroptotic damage (10, 11). Accordingly, ferroptosis has been implicated as an important mechanism underlying vascular stiffening and structural remodeling. Notably, emerging evidence suggests that cellular susceptibility to ferroptosis itself exhibits circadian variation, implying regulation by the molecular clock (12, 13). Although circadian disruption, oxidative stress, and ferroptosis have each been independently associated with hypertension, a key question remains unresolved: how does circadian misalignment translate temporal imbalance into structural damage, thereby driving BP dysregulation.

This review comprehensively summarizes the regulation of circadian rhythms, the molecular mechanisms of ferroptosis, and their respective roles in vascular and renal pathophysiology, and further examines the potential crosstalk between these processes. By integrating existing studies and emerging molecular mechanistic evidence, we identify multiple potential links between circadian disruption and processes related to redox homeostasis, iron metabolism, and ferroptosis. These converging lines of evidence suggest that circadian disruption may disturb the temporal coordination between oxidative stress generation and antioxidant defense and may, in turn, influence the susceptibility of vascular and renal tissues to ferroptosis, potentially contributing to vascular dysfunction, renal injury, and the pathophysiology of hypertension. Based on these lines of evidence and our integrative analysis, we propose the “circadian–ferroptosis axis” as an integrative and testable mechanistic framework to describe the potential pathophysiological interplay between circadian dysregulation and ferroptosis, rather than as an established single causal pathway. On this basis, we further explore the potential therapeutic implications of time-dependent strategies targeting this proposed axis for the management of hypertension.

## 2 Circadian control of BP

### 2.1 Circadian regulation of BP rhythms

The mammalian circadian system is organized in a hierarchical manner, consisting of a central pacemaker located in the suprachiasmatic nucleus (SCN) of the hypothalamus and peripheral clocks distributed across multiple organs (14). The SCN integrates photic input from the retina and synchronizes peripheral oscillators through neural, hormonal, and metabolic cues, thereby maintaining systemic temporal homeostasis (15, 16).

At the cellular level, the mammalian circadian clock is governed by an evolutionarily conserved transcription–translation feedback loop, which generates self-sustained oscillations with an approximately 24-hour period (4). At the core of this molecular oscillator, the transcription factors BMAL1 (also known as ARNTL) and CLOCK form heterodimers that bind to E-box elements within promoter regions, thereby activating the transcription of numerous clock-controlled genes, including members of the Period (PER1-3) and Cryptochrome (CRY1-2) families (17). Following translation in the cytoplasm, PER and CRY proteins accumulate, form repressive complexes, and translocate back into the nucleus, where they inhibit BMAL1-CLOCK transcriptional activity, establishing a negative feedback loop that determines circadian periodicity (17). Oscillatory stability and amplitude are further reinforced by auxiliary regulatory loops. Among these, retinoic acid receptor-related orphan receptors (RORs) and REV-ERB nuclear receptors competitively bind to ROR response elements (ROREs) within the BMAL1 promoter, exerting opposing transcriptional effects: RORs enhance BMAL1 transcription, whereas REV-ERBs repress it (18–20). This secondary stabilizing loop ensures robust rhythmic expression of core clock components (Figure 1).

**Figure 1:**
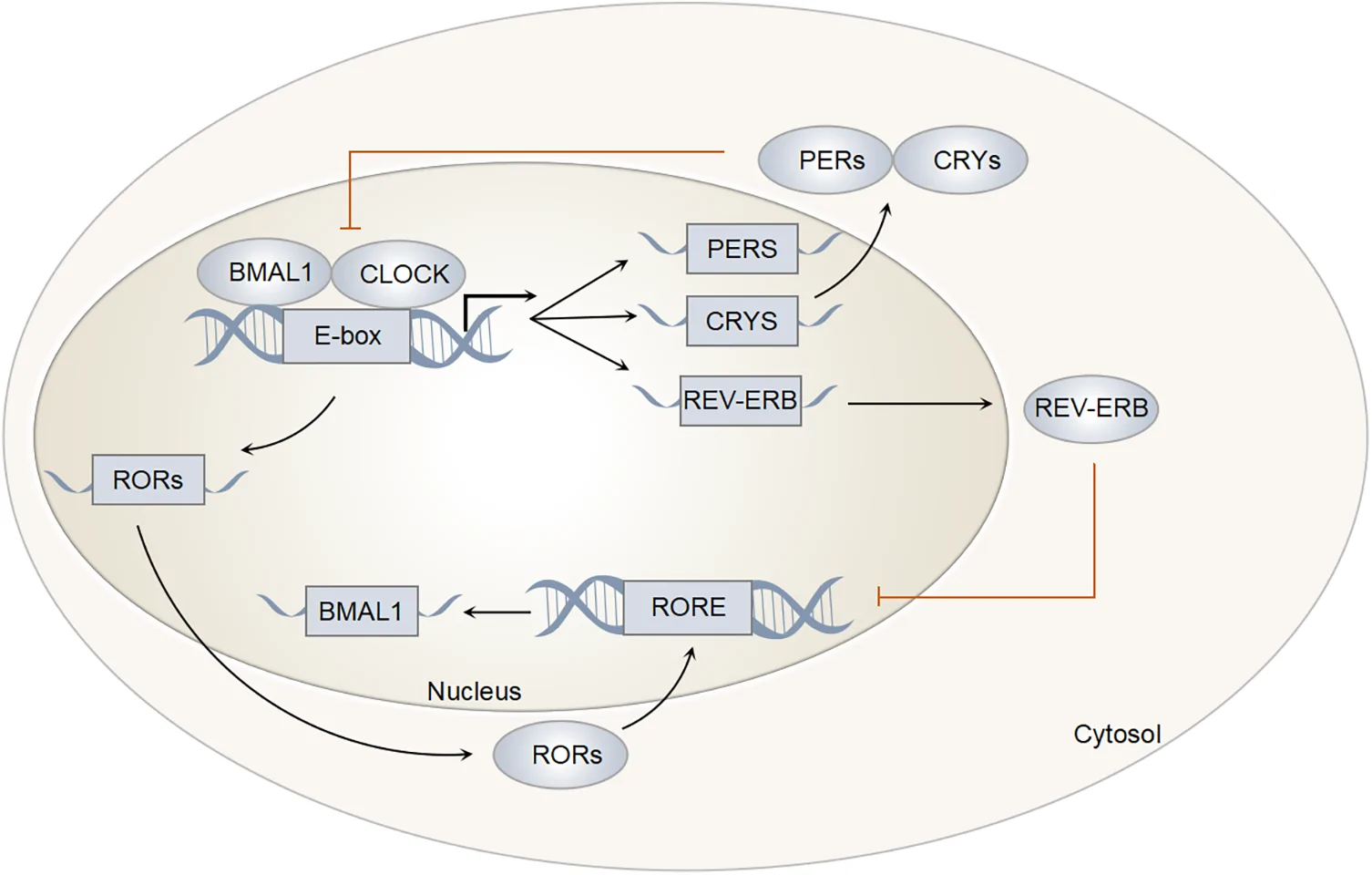
Molecular architecture of the circadian clock. The mammalian circadian clock is organized as an evolutionarily conserved transcription–translation feedback loop that generates ∼24-hour rhythmic oscillations. At the core of this system, BMAL1 and CLOCK form heterodimers that bind to E-box elements in target gene promoters, driving the transcription of key clock genes, including PERs and CRYs. Following cytoplasmic translation, PER and CRY proteins accumulate, assemble into inhibitory complexes, and translocate into the nucleus, where they repress BMAL1-CLOCK-mediated transcription, forming the primary negative feedback loop. In addition, auxiliary regulatory loops further stabilize circadian oscillations: ROR nuclear receptors activate, whereas REV-ERB represses, BMAL1 transcription through competitive binding to ROREs within its promoter. Together, these interlocked feedback circuits ensure the robustness, precision, and amplitude of circadian gene expression.

Beyond sustaining intrinsic rhythmicity, circadian rhythms orchestrate the rhythmic expression of a substantial fraction of the genome, thereby governing diverse physiological processes (4). Notably, numerous clock-controlled genes are implicated in redox homeostasis, iron metabolism, and antioxidant defense systems. Such temporal coordination enables cells to dynamically adapt to diurnal fluctuations in oxidative stress (21, 22). Accordingly, disruption of circadian rhythms compromises antioxidant capacity and facilitates the accumulation of oxidative stress, particularly in metabolically active tissues such as the vasculature and kidneys.

### 2.2 Nighttime as a vascular protective window

Endogenous circadian rhythms and external behavioral factors interact dynamically in a complex and non-linear manner to jointly shape the rhythmicity of BP (23, 24). The normal nocturnal decline in BP, typically ranging from 10% to 20% and associated with sleep and physical inactivity, characterizes individuals classified as “dippers” (25–27). In contrast, those exhibiting a blunted nocturnal decline (<10%) are defined as “non-dippers,” whereas individuals with a paradoxical nocturnal increase in BP are referred to as “reverse dippers” (28–30). Following the nocturnal nadir, BP rises rapidly during the early morning period and upon awakening, a phenomenon commonly described as the morning BP surge (25, 27) (Figure 2).

**Figure 2:**
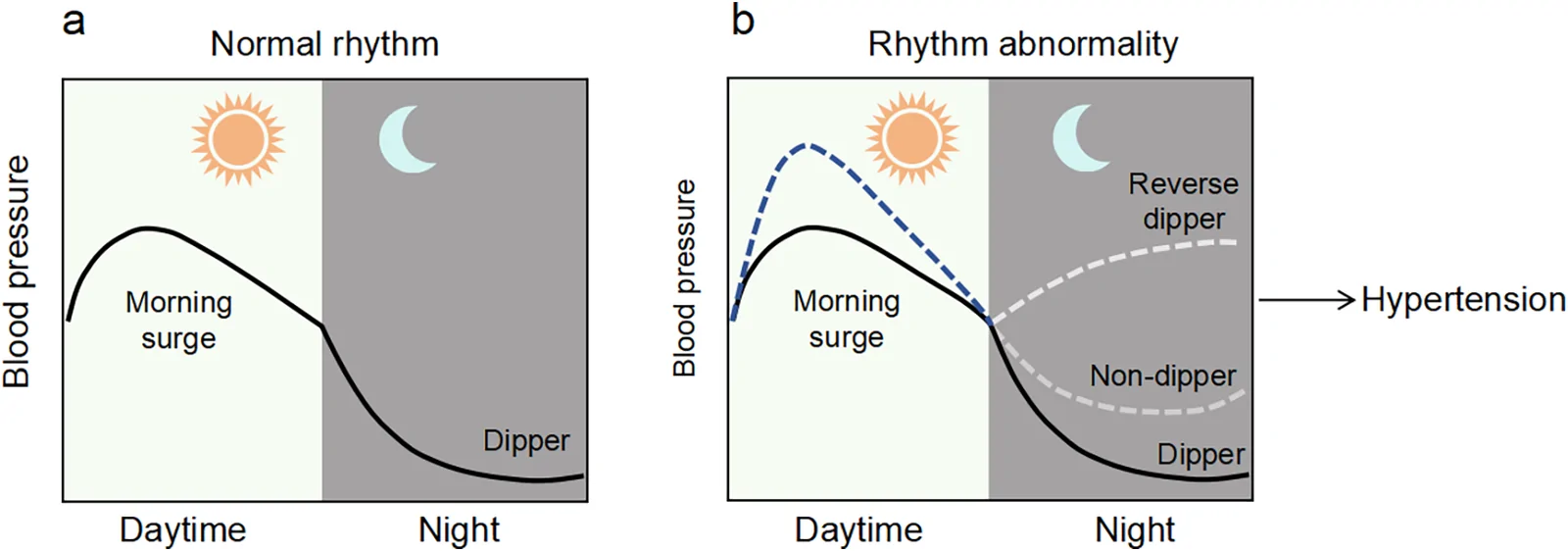
Circadian regulation of BP and patterns of rhythm disruption. (a) Under physiological conditions, BP exhibits a robust circadian rhythm characterized by higher levels during daytime activity and a nocturnal decline during sleep. This normal pattern, termed the “dipper” profile, typically involves a 10–20% reduction in nighttime BP, along with a transient increase upon awakening known as the morning surge. **(b)** Disruption of circadian and behavioral regulation leads to abnormal BP patterns, including “nondipper” (blunted nocturnal decline) and “reverse dipper” (paradoxical nocturnal elevation) phenotypes. These altered rhythms are often accompanied by exaggerated morning surges and are associated with an increased risk of sustained hypertension.

During nocturnal sleep, the decline in arterial pressure is accompanied by reduced mechanical stress on the vascular wall, attenuation of sympathetic nervous system activity, increased melatonin secretion, and an elevation in circulating endothelial progenitor cells (EPCs) (3, 31, 32). Collectively, these alterations shift the vascular physiological state toward anti-oxidative, anti-inflammatory, and reparative processes, thereby contributing to the maintenance of vascular elasticity (33, 34). At the molecular level, core clock proteins, including BMAL1 and CLOCK, coordinate redox homeostasis through transcriptional regulation of antioxidant pathways (35).

From this protective perspective, nocturnal hypotension provides an effective temporal window for vascular repair. If chronic circadian disruption compromises this critical recovery phase, the vascular system may become more susceptible to cumulative mechanical and oxidative injury, thereby accelerating the progression of atherosclerosis and promoting the development of hypertension and other cardiovascular diseases (36). Therefore, it is necessary to delineate the potential links between oxidative stress-mediated damage under circadian disruption and the underlying etiologies of hypertension.

### 2.3 Circadian regulation of redox homeostasis

Gil et al. (37) conducted a comprehensive 24-hour assessment of muscle and plasma in mice with skeletal muscle–specific mitochondrial respiratory uncoupling and demonstrated that mitochondrial dysfunction elicits an integrated stress response (ISR) under circadian control. Specifically, ISR-associated transcriptional activity in skeletal muscle progressively increased during the active phase (night), followed by peak circulating levels of ISR-induced myokines, including FGF21 and GDF15, in the early rest phase. Notably, although mitochondrial dysfunction is typically accompanied by enhanced oxidative stress and an elevated risk of lipid peroxidation, no marked exacerbation of cell death phenotypes was observed. This apparent paradox may be attributable to compensatory upregulation of antioxidant defense systems: the activity of key antioxidant enzymes, such as GPX and NQO1, reached peak levels from the late active phase to the early rest phase, thereby effectively suppressing reactive oxygen species (ROS)-mediated lipid peroxidation. Indeed, previous studies have also demonstrated that cellular antioxidant defense capacity is dynamically regulated by circadian rhythms. Once the temporal synchronization between antioxidant capacity and oxidative stress is disrupted, cells become highly susceptible to oxidative damage resulting from redox imbalance (5, 6).

## 3 Circadian regulation of ferroptosis in hypertension

### 3.1 Molecular mechanisms of ferroptosis

Ferroptosis is an iron-dependent form of regulated cell death characterized primarily by uncontrolled lipid peroxidation (7). Since its initial conceptualization in 2012, the molecular mechanisms underlying ferroptosis have been progressively refined, with increasing attention directed toward identifying its intracellular origins and potential therapeutic implications for refractory diseases (38). Early studies demonstrated that excessive accumulation of mitochondrial ROS triggers peroxidation of membrane phospholipids, initiating free radical chain reactions that ultimately compromise membrane integrity. Subsequent investigations further indicated that the initiating lipid peroxidation cascade may arise from lysosomal iron pools, where redox-active iron accelerates rapid phospholipid oxidation (39). Notably, progressive membrane destabilization ultimately activates a series of downstream events, including NINJ1–mediated plasma membrane rupture. Cells possess multiple antioxidant systems that counteract lipid peroxidation (40, 41). Among these, the glutathione peroxidase 4 (GPX4) pathway functions as a central protective mechanism. GPX4 utilizes GSH to detoxify lipid peroxides and maintain intracellular redox homeostasis (42). The cystine/glutamate antiporter system Xc⁻, a heterodimer composed of SLC7A11 and SLC3A2, mediates cystine uptake in exchange for intracellular glutamate, thereby supplying the substrate required for GSH biosynthesis (43). Meanwhile, ferroptosis suppressor protein 1 (FSP1) acts independently of GPX4 by regenerating lipid-soluble antioxidants, forming a complementary protective axis against ferroptotic damage (44) (Figure 3).

**Figure 3:**
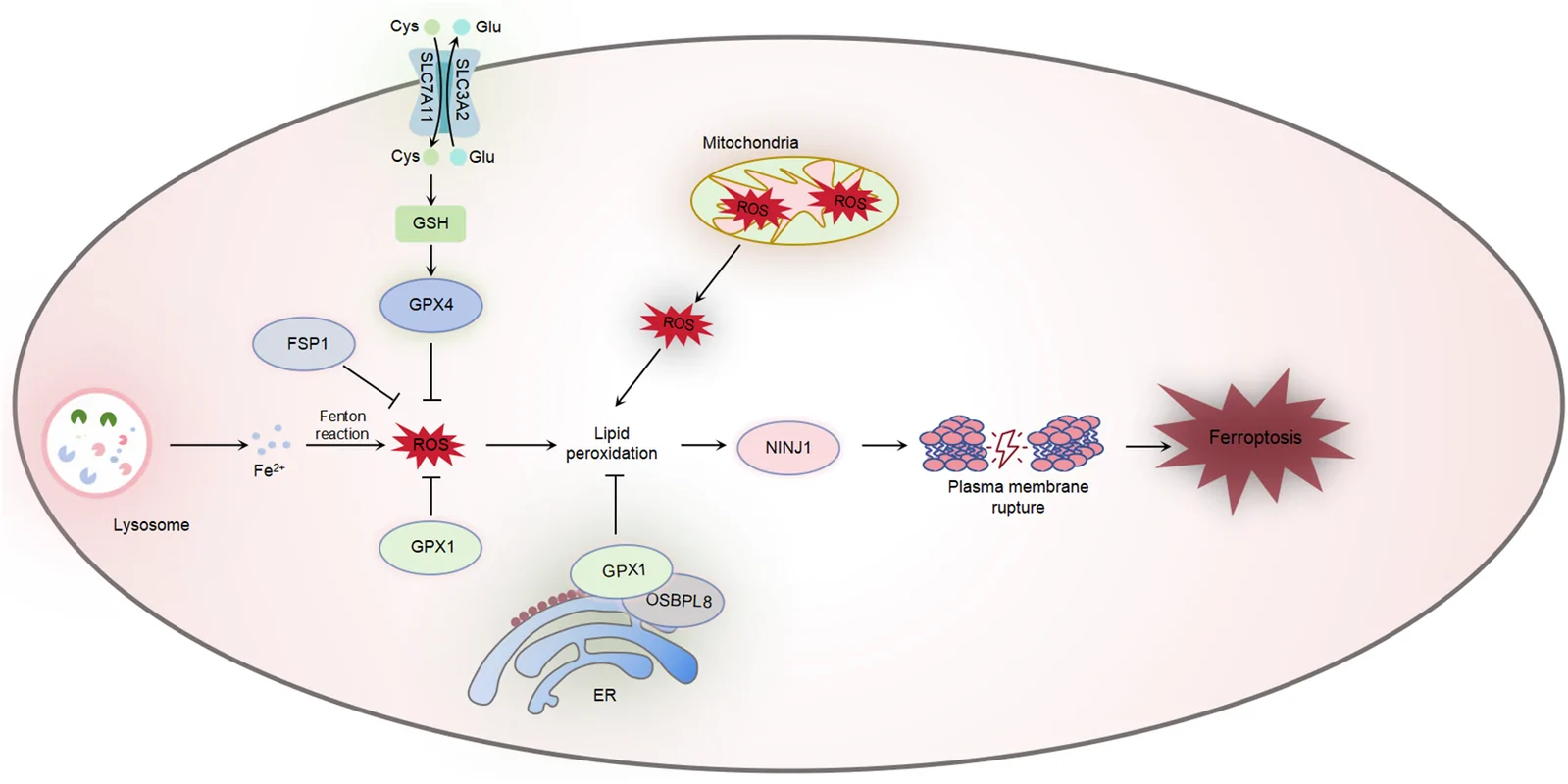
Molecular mechanisms of ferroptosis. Lysosomal iron pools promote the generation of ROS via the Fenton reaction, thereby cooperatively initiating phospholipid peroxidation. Uncontrolled lipid peroxidation cascades subsequently activate NINJ1, ultimately leading to plasma membrane rupture and ferroptotic cell death. The system Xc⁻ antiporter mediates cystine uptake for GSH biosynthesis, while GPX4 utilizes GSH to detoxify lipid peroxides and maintain redox homeostasis. Meanwhile, FSP1 functions independently of GPX4 by regenerating lipid-soluble antioxidants, thereby inhibiting lipid peroxidation. In addition, GPX1 is recruited to the ER by OSBPL8, where it limits ROS-induced lipid peroxidation.

Most mechanistic studies of ferroptosis have relied on pharmacological induction, such as erastin treatment, RSL3 exposure, or direct inactivation of GPX4 (38). However, emerging evidence suggests that ferroptosis may also occur spontaneously under conditions of endogenous oxidative stress. Specifically, recent studies have shown that GPX1 interacts with the cofactor OSBPL8, facilitating its recruitment to the endoplasmic reticulum (ER) where it mitigates ROS-induced lipid peroxidation (45). Loss of GPX1 or OSBPL8 disrupts this protective mechanism, allowing persistent intracellular ROS to trigger ferroptosis even in the absence of exogenous inducers (Figure 3). Such endogenous ferroptotic processes may function as intrinsic quality-control mechanisms that eliminate damaged cells. Importantly, cellular susceptibility to both exogenous and endogenous ferroptotic stimuli is not constant but is dynamically influenced by intracellular metabolic states. Emerging evidence suggests that circadian rhythm-associated redox oscillations may represent an additional regulatory layer that contributes to temporal variation in ferroptosis susceptibility.

### 3.2 Circadian regulation of ferroptosis susceptibility

Emerging evidence suggests that circadian clock components serve as important modulators of ferroptosis susceptibility, rather than merely passive oscillators of metabolic rhythms, thereby conferring pronounced temporal variability in cellular vulnerability to ferroptotic stress. He et al. (46) reported that chronic disruption of circadian rhythms can induce DNA damage and ferroptosis in the spleen of mice. Specifically, compared with age-matched wild-type (WT) mice, 14-month-old Per1/Per2 double-knockout mice exhibited significantly increased splenic lymphocyte death, elevated levels of malondialdehyde (MDA) and ROS, as well as upregulated expression of transferrin receptor 1 (TFR1). Moreover, pro-ferroptotic genes, including Cd36, Atm, and Acsl4, were markedly upregulated in splenic cells of Per1/Per2-deficient mice, whereas anti-ferroptotic genes such as GPX4 were downregulated. In addition, Fan et al. (47) demonstrated that testosterone deficiency–induced downregulation of the circadian clock protein BMAL1 promotes hepatocellular ferroptosis, thereby exacerbating high-fat diet–induced nonalcoholic fatty liver disease (NAFLD). Furthermore, Li et al. (48) reported that aberrant upregulation of CRY2 in trophoblasts from women with recurrent miscarriage suppresses BMAL1 expression, leading to decreased expression of GPX4, xCT, and SOD2, which in turn induces ferroptosis and inhibits trophoblast epithelial–mesenchymal transition (EMT), ultimately contributing to miscarriage. Collectively, these findings suggest that disruption of circadian clock networks may increase ferroptosis susceptibility across multiple tissues. Notably, these observations raise the possibility that similar interactions may also occur in vascular systems, where redox imbalance and iron-dependent lipid peroxidation have been implicated in endothelial and smooth muscle cell injury. Thus, circadian dysregulation may potentially influence ferroptosis-related vascular injury and, in turn, contribute to the pathophysiology of hypertension, although this proposed link remains to be directly established.

### 3.3 Vascular ferroptosis linking circadian disruption to hypertension

Peripheral molecular clocks are widely distributed across multiple cardiovascular cell types, particularly vascular ECs and vascular smooth muscle cells (VSMCs) (49). ECs form a continuous monolayer lining the luminal surface of blood vessels and are directly exposed to hemodynamic shear stress generated by blood flow, thereby serving as a important cellular interface for sensing and transducing biomechanical signals (50). Meanwhile, the intrinsic circadian machinery of VSMCs has been recognized as a important determinant in the establishment of BP rhythmicity. Previous studies have shown that smooth muscle–specific deletion of the core clock gene BMAL1 attenuates the time-of-day–dependent vascular responsiveness to vasoconstrictive stimuli such as norepinephrine and serotonin, resulting in a reduced amplitude of diurnal oscillations in vascular contractility and a blunted synchronization of BP rhythms (51, 52).

Previous experimental studies have demonstrated that shear stress can induce ROS generation in endothelial cells, implicating altered redox signaling in the endothelial response to biomechanical stimuli (53). Under conditions of circadian disruption, such as those associated with sleep disturbances or pathological states, persistent alterations in vascular tone and blood flow may result in abnormal shear stress patterns. It is therefore plausible that circadian disruption could influence endothelial redox homeostasis through altered hemodynamic signaling, although this potential link remains to be directly established. Experimental evidence from the mouse liver has shown that multiple antioxidant defense genes, such as GPX4, are under stringent circadian regulation; disruption of these rhythms results in their downregulation, thereby compromising the detoxifying capacity of the GSH-GPX4 axis (46). Under such conditions, the progressive accumulation of lipid peroxidation products may exceed cellular antioxidant capacity and potentially promote ferroptosis.

Moreover, previous studies have shown that excessive oxidative stress can impair endothelial nitric oxide synthase (eNOS) function and reduce the bioavailability of the vasodilator nitric oxide (NO), while promoting endothelial dysfunction and vasoconstrictive signaling (50, 54). Given the established involvement of ferroptosis in oxidative stress and lipid peroxidation, endothelial ferroptosis may potentially contribute to these alterations, although this specific mechanistic link remains to be further established. This imbalance disrupts vascular homeostasis and favors vasoconstriction, thereby contributing to increased peripheral vascular resistance. The resulting increase in BP may further exacerbate endothelial injury and oxidative stress, potentially creating a self-amplifying vicious cycle (Figure 4). Hypertension and endothelial dysfunction can increase vascular permeability, facilitating the transendothelial entry of low-density lipoprotein (LDL) into the subendothelial space of the arterial wall. Under conditions of oxidative stress, LDL is susceptible to oxidative modification, generating bioactive oxidized LDL (ox-LDL), which can accumulate within the arterial intima (55). Notably, under conditions of excessive oxidative stress, VSMCs can undergo phenotypic switching toward foam cell–like states, which may enhance lipid uptake and deposition; concurrently, oxidative stress can promote their transition toward an osteogenic-like phenotype, thereby contributing to the development and progression of vascular calcification (56, 57) (Figure 4). These phenotypic changes represent forms of cellular remodeling and should be distinguished from ferroptotic cell death.

**Figure 4:**
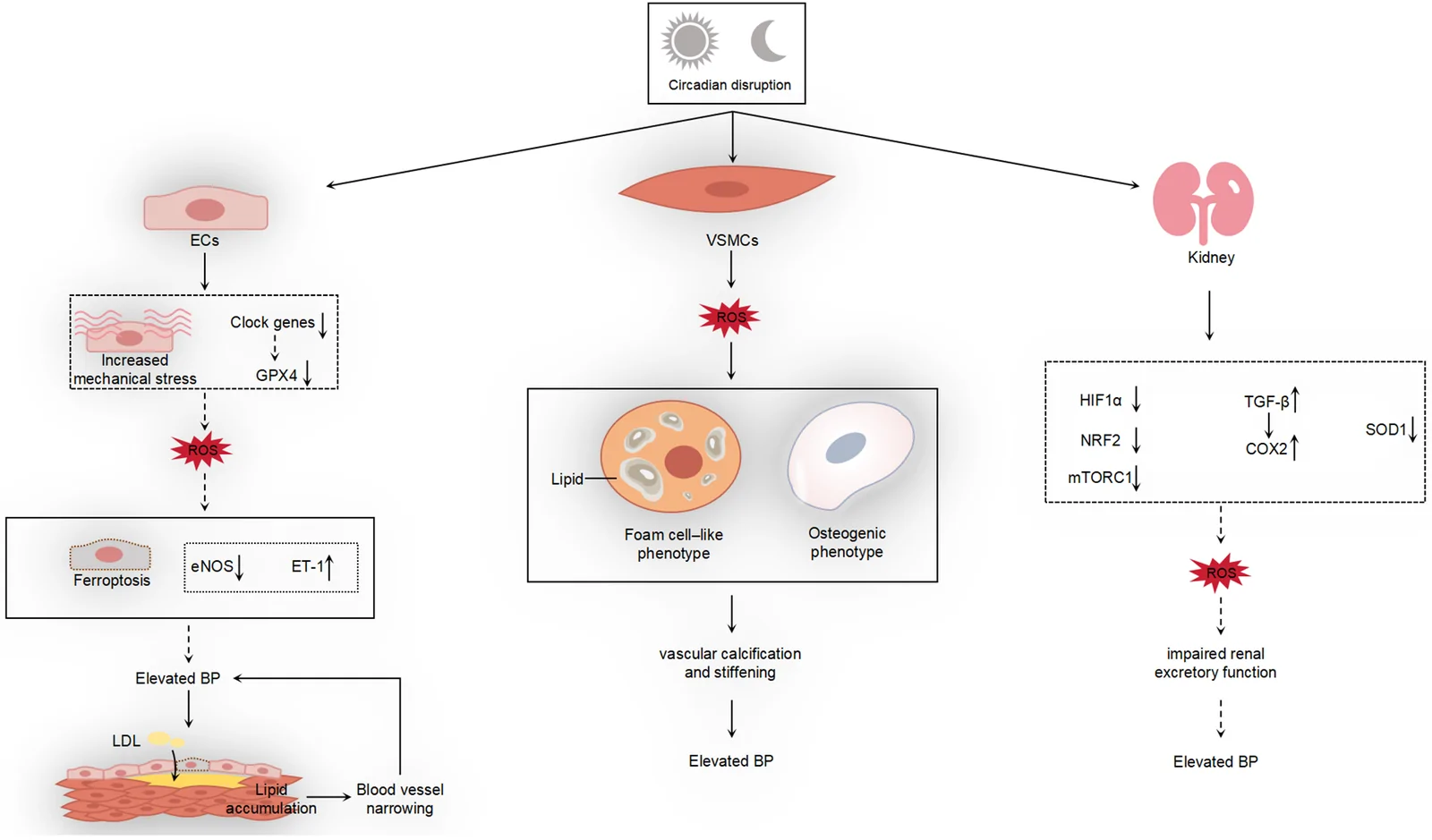
Circadian disruption promotes ferroptosis-mediated vascular and renal injury, thereby increasing the risk of hypertension. Circadian disruption induces ferroptotic damage in vascular and renal tissues through multiple mechanisms. In ECs, elevated mechanical stress promotes mitochondrial ROS production, while circadian disruption downregulates antioxidant genes such as GPX4, leading to excessive ROS accumulation and ferroptosis. Increased ROS further suppresses eNOS expression and activity and enhances ET-1 expression, resulting in vasoconstriction and elevated BP. Sustained hypertension increases vascular permeability, facilitating LDL infiltration into the subendothelial space and contributing to vascular narrowing, which further exacerbates BP elevation. In VSMCs, impaired antioxidant capacity caused by circadian disruption leads to ROS accumulation, driving phenotypic switching toward foam cell–like phenotype and osteogenic phenotype, thereby promoting vascular calcification and stiffening and contributing to hypertension. In the kidney, circadian disruption induces oxidative stress by reducing the expression of multiple antioxidant-related proteins, impairs renal excretory function, and ultimately increases the risk of hypertension.

Circadian regulation also extends to immune-cell recruitment, inflammatory signaling, and inflammasome activation, providing a potential link between circadian disruption and ferroptosis-associated vascular injury (58). Recent evidence indicates that the circadian clock regulates macrophage inflammatory activity through multiple mechanisms. Rev-erb*α* directly represses Ccl2 expression in macrophages, thereby limiting CCL2-dependent ERK/p38 signaling, macrophage adhesion, and migration (59). In parallel, BMAL1 regulates Nrf2 transcription and NRF2-dependent antioxidant responses, such that loss of BMAL1 increases oxidative stress and enhances IL-1*β* production (60). Consistent with this broader role of the molecular clock in inflammatory regulation, time-of-day control of mitochondrial function has been shown to regulate NLRP3 inflammasome activation in macrophages. Macrophages isolated at CT12 exhibited higher mitochondrial membrane potential and greater NLRP3 activation than those isolated at CT0, whereas myeloid-specific Bmal1 deletion enhanced NLRP3 inflammasome activity. Pharmacological disruption of mitochondrial membrane potential attenuated this time-dependent inflammasome activation, indicating that mitochondrial function represents an important intermediate through which the circadian clock modulates inflammatory responses (61). These findings indicate that circadian disruption may alter both the redox state and inflammatory responsiveness of immune cells. Importantly, inflammatory signaling can be propagated from immune cells to the vascular endothelium. TNF-*α* secretion exhibits circadian variation and is influenced by BMAL1-dependent regulation and rhythmic TLR4 signaling, whereas disruption of Bmal1 alters TNF-*α* rhythmicity and enhances inflammatory responses (62, 63). TNF-*α* subsequently activates NF-*κ*B signaling and promotes the transcription of endothelial adhesion molecules, including ICAM1 and VCAM1, thereby facilitating leukocyte adhesion and endothelial activation and contributing to vascular inflammation and injury (64, 65). Together, these findings suggest that circadian disruption may promote vascular inflammation through coordinated alterations in leukocyte recruitment, cytokine and inflammasome signaling, and endothelial activation. Although these studies do not directly establish that circadian inflammatory dysregulation causes vascular ferroptosis, the resulting inflammatory and oxidative microenvironment may increase vascular-cell susceptibility to ferroptotic injury, providing an additional potential link between circadian disruption and ferroptosis-associated vascular damage.

In summary, circadian disruption may contribute to EC and VSMC dysfunction through multiple mechanisms, potentially exacerbating vascular pathology. Over time, these alterations may promote vascular wall remodeling and reduce vascular compliance, which may in turn increase susceptibility to the development and progression of hypertension.

### 3.4 Circadian regulation of renal redox homeostasis and ferroptosis

Previous studies have documented pronounced circadian variation in renal function, as reflected by diurnal differences in urine output (66). Previous experimental studies have demonstrated that renal tissue oxygenation also exhibits significant circadian fluctuations and is closely associated with renal plasma flow and nutrient delivery, suggesting that renal metabolic processes may be subject to circadian regulation (67). Experimental studies in mice have demonstrated that rhythmic oxygen levels can regulate HIF-1*α* activity and reset circadian clocks (68). Given the pronounced circadian variation in renal oxygenation, this oxygen–HIF-1*α* signaling mechanism may also contribute to the regulation of the renal molecular clock, although this possibility remains to be directly established. Moreover, experimental evidence from nucleus pulposus cells has shown that HIF-1*α* can suppress ferroptosis by promoting SLC7A11 translation through YTHDF1 (69). Given the established roles of SLC7A11 and GPX4 in limiting lipid peroxidation, these findings raise the possibility that circadian variation in HIF-1*α* signaling may also influence ferroptosis susceptibility in renal cells, although this potential mechanism remains to be directly investigated. Experimental evidence from human osteosarcoma cells has shown that hypoxia can disrupt circadian oscillations through intracellular acidification and suppression of mTORC1 activity (70). Under hypoxic conditions, metabolic reprogramming and lactate accumulation promote intracellular acidification, which inhibits mTORC1 activity and reduces the translation of circadian clock-related proteins, thereby disrupting circadian oscillations (70). As a central regulator of protein translation and metabolic homeostasis, mTORC1 activity has also been linked to the regulation of ferroptosis-related antioxidant defenses. In glioblastoma cells, inhibition of the AKT/mTORC1 pathway reduces GPX4 expression and promotes ferroptosis (71). These findings raise the possibility that hypoxia-associated suppression of mTORC1 may similarly impair GPX4-dependent antioxidant capacity and increase ferroptosis susceptibility in renal cells, although this proposed mechanism remains to be directly established.

Yang et al. (72) demonstrated that renal NRF2 expression exhibits circadian oscillation under clock control, thereby regulating the antioxidant capacity and ferroptosis sensitivity of tubular epithelial cells. During periods of reduced NRF2 activity, exposure to contrast agents exacerbates lipid peroxidation and ferroptosis, leading to more severe renal injury; conversely, pharmacological activation of NRF2 effectively suppresses ferroptosis and mitigates kidney damage. Notably, HIF-1*α*, NRF2, and mTORC1 have each been implicated in the regulation of oxidative stress and ferroptosis. Their potential convergence may therefore represent an important mechanism linking circadian dysregulation to ferroptotic injury in the kidney; however, whether these pathways function as a coordinated and synergistic regulatory network in renal ferroptosis remains to be directly established (Figure 4).

In addition, the circadian system aggravates renal injury by modulating other oxidative stress–related pathways. Under physiological rhythmic conditions, the CLOCK–BMAL1 heterodimer suppresses the TGF-*β*-COX2 profibrotic signaling pathway, thereby alleviating oxidative stress and limiting tissue damage. In contrast, in clock-deficient mouse models, the loss of this circadian protective mechanism results in a marked upregulation of TGF-*β*-mediated COX2 expression in the unilateral ureteral obstruction model, which in turn exacerbates oxidative stress and accelerates renal fibrogenesis (73). The same study further demonstrated that SOD1 expression exhibits robust circadian oscillation in the kidney, whereas CLOCK deficiency abolishes this rhythmicity and significantly reduces SOD1 expression in mice with or without UUO surgery (73). In contrast, SOD2 expression showed neither significant circadian oscillation nor significant differences between wild-type and CLOCK-deficient mice (65). These findings suggest that circadian disruption–associated downregulation of SOD1 may represent an important mechanism contributing to increased oxidative stress in the kidney.

Collectively, these findings suggest that circadian disruption may impair the expression of multiple antioxidant-related genes in renal cells, potentially weakening cellular antioxidant defenses and increasing susceptibility to ferroptosis. Renal ferroptotic injury may, in turn, compromise tubular reabsorptive and excretory functions, potentially affecting sodium and water homeostasis and the regulation of BP rhythmicity. These alterations may contribute to the development and progression of hypertension, although the extent to which ferroptosis directly mediates this circadian–renal–BP link remains to be established.

### 3.5 Interactions between ferroptosis and established hypertensive pathways

Ferroptosis may represent an interconnected component of the broader pathophysiological network underlying hypertension rather than an isolated pathogenic mechanism. Activation of the renin–angiotensin–aldosterone system (RAAS), particularly excessive angiotensin II (Ang II) signaling, can promote vascular oxidative stress and lipid peroxidation, processes that have been directly implicated in endothelial ferroptosis and vascular dysfunction (74). Recent evidence from a stress-induced hypertension model showed increased mitochondrial oxidative stress and ferroptosis in neurons of the rostral ventrolateral medulla (RVLM), a key brainstem region controlling sympathetic outflow. Resveratrol activated the AMPK/Sirt3 pathway, reduced RVLM oxidative stress and ferroptosis, suppressed sympathetic activity, and ultimately lowered blood pressure. These findings suggest that ferroptosis-related neuronal injury may contribute to sympathetic overactivity and hypertension, although whether sympathetic activation directly promotes ferroptosis in peripheral vascular or renal tissues remains to be established (75).

## 4 Therapeutic targeting of the circadian–ferroptosis axis in hypertension

Emerging evidence suggests that ferroptosis in vascular and renal cells may contribute to the link between circadian disruption and hypertension. Accordingly, therapeutic strategies targeting the circadian rhythm-ferroptosis axis may provide novel opportunities for BP control.

### 4.1 Mechanistic evidence from non-hypertensive models

Although direct evidence linking ferroptosis to BP control remains limited, studies in non-hypertensive models provide mechanistic support for the interaction between circadian regulation, ferroptosis, and renal or vascular injury. Renal injury is closely associated with abnormal circadian BP patterns, and accumulating evidence suggests that alleviating ferroptosis in renal tissues under conditions of circadian disruption may help reduce BP fluctuations secondary to impaired renal function. Furthermore, Zhou et al. (76) demonstrated that hyperoxaluria impairs mitophagy, leading to excessive accumulation of ROS, which in turn induces ferroptosis in renal tubular epithelial cells and promotes kidney stone formation. Melatonin exerts a protective effect against kidney stone formation by activating AMPK/PINK1-Parkin-mediated mitophagy and inhibiting ferroptosis in renal tubular epithelial cells. In addition, the core circadian gene BMAL1 has been shown to regulate NRF2 and its downstream antioxidant signaling pathways, thereby influencing the time-dependent features of ferroptosis in renal transplantation and associated ischemia–reperfusion injury. Accordingly, identifying critical temporal windows during which the kidney is more susceptible or relatively resistant to injury, and optimizing the timing of surgical interventions or implementing targeted protective strategies based on these windows, may represent an important approach to mitigate tissue damage (77). Furthermore, sepsis disrupts circadian rhythms and leads to renal dysfunction. In a mouse model of sepsis-associated acute kidney injury (SA-AKI), increased expression of NFIL3 in renal tubular cells promotes ferroptosis and inflammatory responses through upregulation of ACSL4, ultimately resulting in renal dysfunction (78). Therefore, targeting the NFIL3/ACSL4 axis may represent a promising therapeutic strategy for the treatment of sepsis-associated acute kidney injury.

### 4.2 Preclinical evidence in hypertension

Preclinical evidence directly linking ferroptosis to blood pressure control has emerged from several experimental models of hypertension. Previous studies have identified PPAT and FXR1 as molecular regulators involved in the establishment of renal circadian BP rhythms. Core clock genes, such as BMAL1 and CLOCK, regulate the expression of PPAT and FXR1, thereby influencing the formation of renal circadian BP control mechanisms. Notably, in spontaneously hypertensive mice, PPAT expression is significantly upregulated in renal tubular cells; this upregulation may contribute to abnormal BP variability by mediating renal and vascular toxicity associated with altered uric acid metabolism (79). Studies have shown that Sestrin2 overexpression suppresses Ang II-induced ferroptosis in HUVECs, whereas Sestrin2 knockdown promotes ferroptotic responses (80). Sestrin2 overexpression also partially restored normal mitochondrial morphology in these cells. *In vivo*, Sestrin2 overexpression attenuated ferroptosis in Ang II-treated mice and reduced both systolic blood pressure (SBP) and diastolic blood pressure (DBP). Conversely, renal Sestrin2 silencing exacerbated oxidative stress and increased blood pressure in mice (81). Similarly, in D2 receptor-induced hypertensive mice, Sestrin2 overexpression reduced renal oxidative stress and lowered blood pressure (82). Pharmacological inhibition of ferroptosis with ferrostatin-1 (Fer-1) has also been shown to ameliorate Ang II-induced hypertension in mice. Beyond endothelial cells, ferroptosis has been observed in VSMCs, and Fer-1 attenuated high hydrostatic pressure- or Ang II-induced VSMC ferroptosis both *in vitro* and *in vivo* through the SLC7A11/GPX4 pathway (83). Liang et al. (84) demonstrated, using mouse aortic tissues and human umbilical vein endothelial cells, that Lenvatinib-induced inhibition of YAP activity and nuclear translocation was accompanied by reduced GPX4 expression and increased lipid peroxidation, LDH release, and MDA levels, thereby promoting endothelial ferroptosis. Notably, pharmacological inhibition of ferroptosis with Fer-1 markedly improved Lenvatinib-induced endothelium-dependent relaxation dysfunction and hypertension, suggesting that YAP inhibition-mediated endothelial ferroptosis may represent an important mechanism underlying Lenvatinib-associated vascular dysfunction and hypertension. In an Ang II-induced hypertensive mouse model and renal tubular epithelial cells, Li et al. (85) found that SIRT7 alleviated renal ferroptosis, lipid peroxidation, and partial epithelial–mesenchymal transition (EMT) under hypertensive conditions by promoting KLF15/Nrf2 signaling and downstream antioxidant defenses, thereby attenuating renal interstitial fibrosis, structural injury, and renal functional impairment. Importantly, although ferroptosis-targeted interventions have shown BP-lowering effects in several experimental models of hypertension, these studies have primarily assessed endpoint BP or hypertension-associated tissue injury. Whether ferroptosis inhibition can restore abnormal circadian BP patterns, particularly nocturnal dipping, has not yet been directly demonstrated.

### 4.3 Clinical evidence and translational considerations

Clinical evidence remains limited. Although ambulatory blood pressure monitoring (ABPM) has established the prognostic importance of nocturnal BP and abnormal dipping patterns in hypertension, clinical studies directly evaluating ferroptosis-targeted interventions in relation to BP reduction or circadian BP restoration are currently lacking. Similarly, no clinical evidence has yet demonstrated that pharmacological ferroptosis inhibitors can restore the physiological nocturnal BP dipping pattern.

### 4.4 Circadian-based and non-pharmacological approaches

In addition, intermittent fasting (IF), as an intervention closely associated with circadian regulation, has been demonstrated to attenuate ferroptosis by modulating the circadian clock/BMAL1–AMPK/NRF2 axis (86). Specifically, IF restores BMAL1 expression while concomitantly activating AMPK and NRF2 signaling pathways, thereby enhancing antioxidant defense capacity and reducing lipid peroxidation levels. Notably, a bidirectional crosstalk exists between circadian clock components and metabolic redox pathways, enabling coordinated regulation of ferroptosis. These findings suggest that IF may serve as a potential non-pharmacological intervention for mitigating ferroptosis-associated vascular and renal injury. However, evidence directly linking IF-mediated ferroptosis modulation to BP lowering or restoration of circadian BP dipping in hypertension remains insufficient.

### 4.5 Therapeutic implications and remaining gaps

Overall, experimental agents such as ferrostatin-1 have shown protective effects in preclinical models; however, their pharmacokinetic properties, tissue distribution, target specificity, and long-term safety remain insufficiently characterized, limiting their current applicability to chronic antihypertensive therapy. Moreover, because ferroptosis is involved in physiological cellular stress responses, sustained systemic inhibition may potentially produce unintended effects that remain poorly understood. Taken together, current evidence supports circadian modulation and ferroptosis inhibition as two potentially complementary therapeutic strategies, but whether a causal therapeutic relationship exists between these processes has not yet been established in humans. Future studies should therefore integrate mechanistic investigations with well-designed clinical studies to further determine whether ferroptosis represents a modifiable mechanism linking circadian disruption to the development and progression of hypertension and whether targeting this pathway can improve both blood pressure control and its circadian pattern.

## 5 Discussion

Aging of the global population is accelerating, with cardiovascular-related chronic diseases accounting for an increasing proportion of mortality among individuals over 70 years of age (87). Hypertension, as one of the most prevalent cardiovascular disorders, exhibits a marked age-dependent increase in incidence. Emerging evidence has highlighted that sleep disturbances and circadian misalignment exert profound effects on cardiovascular resilience (88). However, a standardized quantitative framework integrating sleep patterns, circadian alignment, and cardiovascular adaptability into a unified predictive model remains lacking. Consistently, ambulatory BP monitoring (ABPM) has revealed that nocturnal systolic BP is a significantly stronger predictor of cardiovascular mortality risk than daytime office measurements, with substantially greater prognostic value (89). ABPM provides a clinically valuable approach for characterizing the temporal dynamics of BP over a 24-hour period and identifying abnormal circadian BP phenotypes. Accordingly, abnormal nocturnal BP patterns may serve not only as clinically relevant indicators of impaired circadian BP regulation but also as measurable phenotypes for investigating the potential biological consequences of circadian disruption. Notably, elevated nocturnal BP reflects a disruption of circadian regulatory mechanisms, resulting in sustained exposure of the vasculature to hemodynamic stress in the absence of adequate restorative phases. Such chronic nocturnal pressure overload may promote subclinical vascular injury, thereby increasing the long-term risk of adverse cardiovascular outcomes (90) (Figure 5). These clinical observations raise an important question regarding which real-world factors contribute to circadian disruption and, consequently, to abnormal BP rhythmicity.

**Figure 5:**
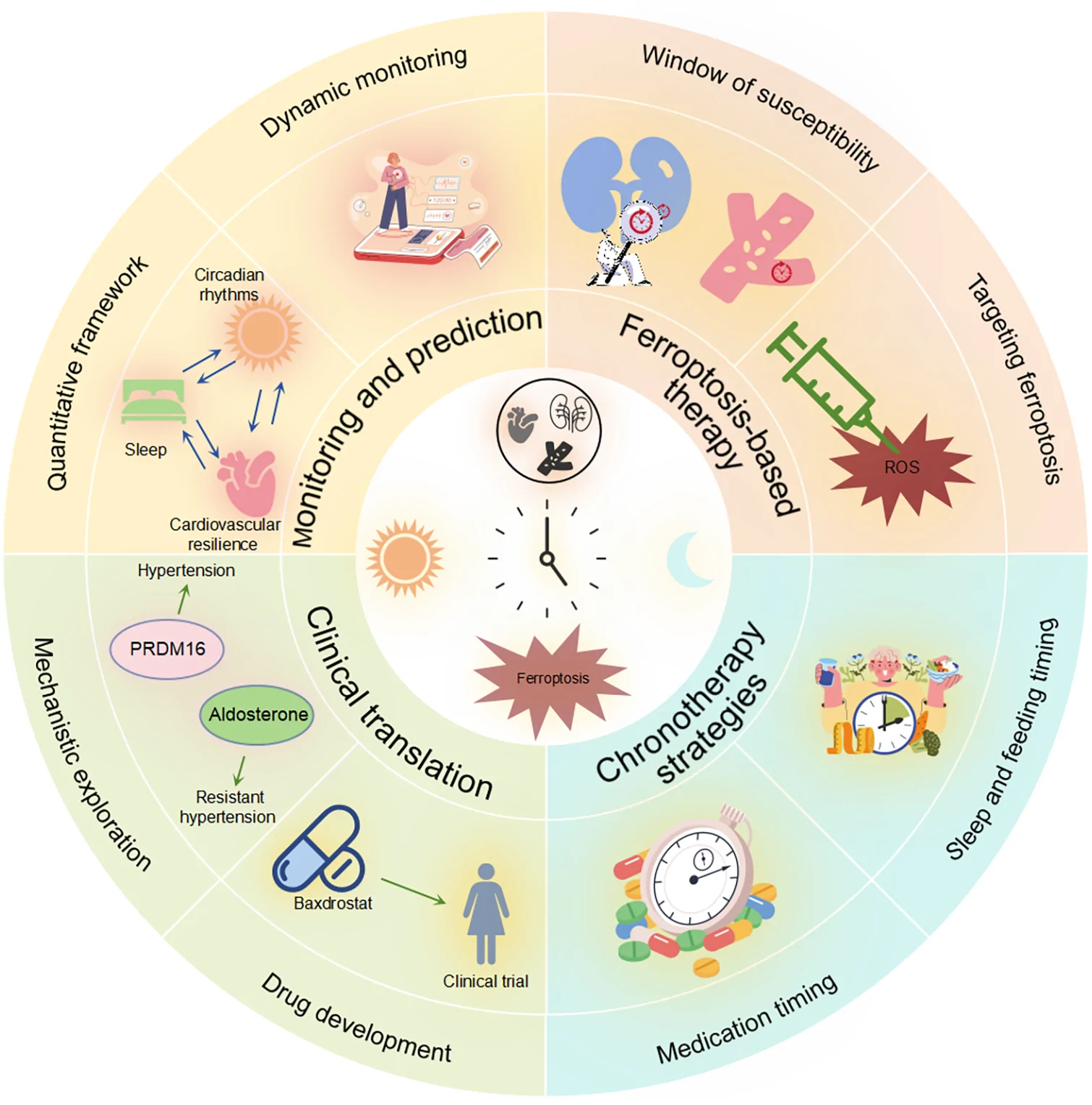
Circadian rhythm–dependent ferroptosis in hypertension: monitoring and prediction,chronotherapy strategies,and clinical translation. Monitoring and prediction approaches, including quantitative frameworks and dynamic monitoring, enable real-time assessment of circadian rhythms and BP variability. Ferroptosis-based therapeutic strategies emphasize the identification of windows of susceptibility and the targeting of ferroptosis to optimize intervention efficacy. Chronotherapy strategies, such as sleep and feeding timing as well as medication timing, aim to restore circadian alignment and improve BP control. Furthermore, clinical translation integrates mechanistic exploration, drug development, and clinical trials to facilitate the application of ferroptosis-related interventions. However, challenges including limited temporal resolution in monitoring, individual variability in circadian responsiveness, and constraints in clinical implementation remain to be addressed for the effective translation of these strategies into hypertension management.

Shift work represents a common real-world condition of circadian disruption and has increasingly been associated with impaired BP regulation. Accumulating evidence indicates that shift work may affect BP regulation in shift workers by disrupting normal circadian physiology (91, 92). Experimental studies in animal models have further demonstrated that dysregulation of circadian clock genes can directly influence BP regulation (93, 94). Although the association between circadian disruption and hypertension has been extensively investigated, the underlying molecular and physiological mechanisms remain to be fully elucidated. Previous studies have reported that night-shift work can disrupt circadian rhythms and impair mitochondrial function (95). Jiang et al. (96) further demonstrated that night-shift work was associated with elevated BP in both human populations and mouse models, an effect that may be mediated by circadian disruption. Notably, this study found that circadian disruption-associated mitochondrial dysfunction and increased oxidative stress may represent important mechanisms linking shift work to elevated BP.

These findings suggest that disruption of circadian organization may alter cellular redox homeostasis, which may in turn provide a potential mechanistic context for increased susceptibility to ferroptotic stress. In addition, emerging evidence suggests that circadian disruption may promote BP elevation through autonomic imbalance or dysregulated sodium retention (97, 98). Vascular compliance in response to volume load, as well as renal sodium and water handling, are fundamental determinants of BP variability. Circadian clock genes orchestrate the rhythmic expression of key regulatory factors in vascular smooth muscle cells and renal tissues, thereby governing physiological oscillations in BP (79, 99). Notably, circadian fluctuations in cellular antioxidant capacity may establish time-dependent “vulnerable windows” (37), during which disruption of redox homeostasis may promote lipid peroxidation and increase susceptibility to ferroptotic stress, potentially contributing to endothelial dysfunction, vascular stiffening, and impaired renal excretory function (36, 100). In this context, the precise identification of temporal windows with heightened ferroptosis susceptibility in vascular and renal tissues, followed by targeted intervention, may represent a promising strategy for hypertension management in the future. Although direct evidence in hypertension remains limited, mechanistic insights have been provided by studies in other disease models. Melatonin has been shown to inhibit ferroptosis by reprogramming the circadian clock protein BMAL1 and subsequently activating the NRF2-ARE antioxidant pathway, thereby alleviating ethanol-induced liver injury (101). In addition, exosomes derived from melatonin-preconditioned bone marrow mesenchymal stem cells can deliver USP4, which mediates the deubiquitination of BMAL1, stabilizing BMAL1 protein levels to restore normal circadian rhythms and suppress ferroptosis in skin flap grafts in sleep-restricted mice, ultimately improving tissue injury and regenerative capacity (102). Furthermore, nobiletin treatment has been reported to activate the Bmal1/NRF2 axis, induce mitophagy, and upregulate GPX4 expression, thereby attenuating ferroptosis and redox imbalance and ameliorating rotenone-induced Parkinson's disease (103) (Figure 5). Collectively, these findings provide mechanistic support for functional crosstalk between circadian regulation and ferroptosis, while their relevance to circadian BP dysregulation in hypertension remains to be determined.

A major challenge for translating this framework to clinical practice is the lack of validated biomarkers that can specifically and dynamically reflect ferroptosis in patients with hypertension. Although lipid peroxidation products such as malondialdehyde and 4-hydroxynonenal, together with ferroptosis-related regulators including GPX4, SLC7A11, ACSL4, and iron-related parameters, have been widely investigated, none can currently be considered a specific clinical indicator of ferroptosis because these signals may also arise from broader oxidative, inflammatory, or tissue-injury processes (104, 105). Thus, rather than relying on a single biomarker, future clinical studies may benefit from integrating multimodal ferroptosis-related signatures with 24-h ambulatory blood pressure monitoring. Such an approach could determine whether time-dependent changes in ferroptosis-related signals are associated with abnormal BP rhythms, particularly nondipping or nocturnal hypertension, and may help identify periods of increased ferroptotic susceptibility. Importantly, establishing such temporal associations would provide a basis for testing whether time-targeted interventions can modify both ferroptosis-related responses and circadian BP patterns, thereby offering a more direct clinical test of the proposed circadian–ferroptosis framework.

With an increasing understanding of the complexity of hypertension pathogenesis, significant advances have been made in recent years in local tissue regulation, systemic endocrine signaling, and their time-dependent integration. Emerging evidence indicates that perivascular adipose tissue (PVAT), which surrounds blood vessels, regulates the underlying vascular tone through the secretion of adipokines. When PVAT formation is impaired due to factors such as PRDM16 deficiency, it may contribute to the development of atherosclerosis and hypertension (106). These findings suggest that targeting PVAT and its derived chemical mediators may provide novel insights and therapeutic targets for the development of future antihypertensive agents. Liang et al. (107) reported that in primary aldosteronism, excessive aldosterone has been shown to attenuate the physiological nocturnal decline in BP, resulting in a non-dipping phenotype. Beyond its well-established roles in sodium retention and volume expansion, aldosterone may directly influence the circadian rhythm of BP. Therefore, the management of primary aldosteronism should not only focus on achieving target BP levels but also on the restoration of normal circadian BP patterns. In addition, dysregulation of aldosterone is a major contributor to resistant hypertension. A recent phase III clinical trial demonstrated that the novel selective aldosterone synthase inhibitor baxdrostat significantly reduced 24-hour ambulatory systolic BP in patients with resistant hypertension, highlighting the therapeutic potential of targeting aldosterone synthase in difficult-to-control hypertension (108). This therapeutic benefit may also involve the restoration of normal circadian BP rhythms through aldosterone suppression (Figure 5).

Emerging evidence further supports time as an underappreciated dimension in the regulation of BP homeostasis. Torimitsu et al. (109) reported that the colon serves as an important peripheral clock organ integrating feeding timing with salt-related signals. When excessive salt is consumed at inappropriate times—particularly during the daytime rest phase—it may not only increase sodium load but also disrupt circadian synchrony within the gut and across the whole body, ultimately leading to impaired BP regulation. This insight provides new perspectives for the prevention and management of hypertension: while attention to total salt intake remains essential, the timing of intake may be equally critical. On this basis, time-based dietary intervention strategies may be developed in the future, aiming to restore regular feeding rhythms and thereby optimize sodium metabolism and BP control. Consistent with this view, behavioral alignment of the circadian clock has also been shown to influence BP regulation. Thosar et al. (110) demonstrated that hypertensive patients who maintained a fixed bedtime over a two-week period exhibited a significant reduction in BP, particularly nocturnal BP. Notably, this intervention remained effective even among participants already receiving antihypertensive medications. These findings suggest that regularization of pre-sleep timing represents a low-cost and low-risk intervention that may serve as a valuable adjunct to existing antihypertensive therapies. In addition, the timing of medication administration has emerged as another critical component of chronotherapy. Ye et al. (111) demonstrated that bedtime administration of a combination therapy of olmesartan and amlodipine, compared with morning dosing, significantly reduced nocturnal systolic BP in hypertensive patients, while improving circadian BP patterns without increasing the risk of nocturnal hypotension (Figure 5). Although these findings suggest that chronotherapy may represent a low-cost and relatively low-risk intervention, its applicability is likely to be limited to patients with specific circadian or blood pressure rhythm disturbances. The implementation of chronotherapy requires consideration not only of individual chronotype, BP rhythmicity, treatment adherence, and the risk of nocturnal hypotension, but also of drug class and pharmacokinetic properties, including drug half-life. In other words, the potential benefit of a given dosing time may vary according to both patient characteristics and drug properties: different patients may derive different benefits from the same drug administered at a given time, while the optimal timing of different antihypertensive agents may also vary within the same individual. A key challenge for future chronotherapy therefore lies in integrating individual circadian phenotypes and BP dynamics with drug-specific pharmacokinetic characteristics to develop more precise and individualized dosing strategies. Chronotherapy should consequently be considered an adjunctive rather than a replacement strategy, complementing pharmacological treatment and other established approaches to hypertension management.

Collectively, the integration of circadian biology and ferroptosis highlights a critical transition from static to time-resolved pathophysiology. Future studies should incorporate circadian timing as a key experimental variable, as oscillations in core anti-ferroptotic regulators may fundamentally influence the reproducibility and interpretation of ferroptosis-related findings. The construction of a 24-hour “ferroptosis susceptibility landscape” through time-series multi-omics and real-time redox imaging may facilitate the identification of temporally defined vulnerability windows across cardiovascular, renal, and other organ systems. Targeting ferroptosis in a mechanism-specific manner at optimal circadian phases may enhance therapeutic efficacy while minimizing off-target effects. Furthermore, integrating computational modeling with experimental and clinical data will be essential to predict time-dependent ferroptosis risk and to guide precision chronotherapy.

## 6 Conclusion

The evidence reviewed establishes that circadian rhythm disruption may impair the redox homeostasis of blood pressure-regulating organs, including the kidney and vasculature, thereby increasing their susceptibility to ferroptosis and ultimately leading to dysregulated blood pressure control. Although most of the current evidence remains correlational or indirect, direct causal evidence demonstrating that circadian dysregulation drives hypertension through ferroptosis remains limited. With the accumulation of further clinical and experimental evidence, targeting the circadian rhythm–ferroptosis axis may emerge as an effective strategy for the prevention and treatment of circadian disruption–associated hypertension.
